# Comprehensive analysis of quality characteristics in main commercial coffee varieties and wild Arabica in Kenya

**DOI:** 10.1016/j.fochx.2022.100294

**Published:** 2022-03-25

**Authors:** Collins Ogutu, Sylvia Cherono, Charmaine Ntini, Lu Wang, Yuepeng Han

**Affiliations:** a CAS Key Laboratory of Plant Germplasm Enhancement and Specialty Agriculture, Wuhan Botanical Garden, The Innovative Academy of Seed Design, Chinese Academy of Sciences, Wuhan 430074 China; b Center of Economic Botany, Core Botanical Gardens, Chinese Academy of Sciences, Wuhan 430074, China; c University of Chinese Academy of Sciences, 19A Yuquanlu, Beijing 100049, China; d Sino-African Joint Research Center, Chinese Academy of Sciences, Wuhan 430074 China

**Keywords:** Arabica, Caffeine, Chlorogenic acid, Trigonelline, Sucrose, Flavor, Aroma

## Abstract

Coffee flavor is a complex commercial trait and its generation mechanisms remain largely unclear. Here, we investigated non-volatile and volatile compounds in the AA grade coffee beans of cultivated and wild *Coffea arabica* accessions in Kenya. An increased accumulation of trigonelline and sucrose along with a decreased accumulation of caffeine and 5-caffeoylquinic acid or chlorogenic acid (CGA) relative to wild Arabica contribute to the improved flavor of commercial varieties. Trigonelline was strongly associated with attributes of coffee brews such as pH, aroma intensity and antioxidant activity, suggesting that it is one of the main flavor precursors. Partial least squares discriminant analysis (PLS-DA) identified 18 volatiles that could potentially define flavor quality of coffee brews, with pyrazines and thiols as the major coffee flavor determinants.

## Highlights

• The contents of key non-volatile compounds, including chlorogenic acid, trigonelline, caffeine, and sucrose, vary significantly among Arabica cultivars in Kenya.
• Trigonelline is strongly associated with quality attributes of coffee brews.
• Pyrazines and thiols are major coffee flavor determinants in commercial cultivars in Kenya.

## 1 Introduction

Coffee beverage quality is determined by a combination of aroma and taste, which are associated with the accumulation of volatile and non-volatile compounds. Accumulation of non-volatile compounds such as caffeine, chlorogenic acid (CGA), trigonelline and sucrose in green coffee beans is influenced by both genetic and environmental factors including altitude, shade and post-harvest processing (Cheng et al., 2016, Vaast et al., 2006, Avelino et al., 2005). Sucrose and trigonelline are associated with high beverage quality, while caffeine acid and chlorogenic are responsible for the bitterness taste of coffee beverage (Toledo et al., 2016). *C. arabica* is characterized by low caffeine and CGA content which ensures its desirable beverage quality. In Kenya, coffee production is mainly from the locally developed high beverage quality *C. arabica* cultivars, ‘R11′, K7′, ‘SL28′ and ‘SL34′, which are grown in varied elevations and environmental conditions. ‘R11′ is a recently developed high yielding, disease resistant hybrid, but it is controversially considered a low cup quality cultivar. By contrast, ‘SL28′, ‘SL34′ and ‘K7′ are high quality traditional cultivars that were developed in early 1900s (Van der Vossen, 2009, Agwanda et al., 2003). In addition, mount Marsabit in the northern region of Kenya harbors valuable wild Arabica germplasm which is still less studied due to previous challenges such as inaccessibility to the mountain area and lack of rigorous and targeted fieldwork.

Commercial green coffee beans in Kenya are mechanically graded by quality as E, PB, AA, AB, C, TT, and T categories based on size, shape and density with no consideration on the variety (Omondi, 2009). The AA grade beans forms the country’s largest coffee market category after post-harvest processing and is considered as one of the world's finest specialty coffees. Despite production decline in recent years, the quality of traditional Kenyan coffee varieties remains highly valued internationally and it falls within the Colombian mild category, which are price competitive, fine Arabica coffee with balanced acidity and pleasant distinctive aroma (Moreno, Moreno, & Cadena, 1995). A narrow genetic diversity exists between cultivated varieties in Kenya and the wild Arabica relatives found in Mt. Marsabit (Ogutu et al., 2016). Marked flavor difference exists between cultivars despite their high genetic similarity, with strong market desirability for ‘SL28′ due to its perceived superior beverage quality (Cheng et al., 2016, Agwanda et al., 2003). However, comparative chemical characterization and comprehensive sensory studies on these coffee accessions is still lacking. In addition, the biochemical fingerprinting of these wild Arabica relatives found in Kenya is limited.

A large number of volatile compounds are generated from degradation of non-volatile compounds such as sucrose, trigonelline, chlorogenic acid and caffeine as well as from a cascade of reactions between free amino acids and reducing sugars through both Maillard and Strecker degradation pathways during coffee bean roasting (Buffo & Cardelli-Freire, 2004). As a result, several classes of volatile compounds such as furans, esters, pyridines, thiols, alcohols, pyrazine, pyrroles, ketones, aldehydes, phenols, lactones, and terpenes have been identified in coffee. However, only a small fraction is crucial for determining the olfactory sensation produced (Sittipod et al., 2019, Charles-Bernard et al., 2005). Aroma is a key organoleptic quality and variation in its composition is responsible for flavor difference in coffee (Dong et al., 2015, Sunarharum et al., 2014). The proportion of volatile compounds varies with processing and storage and is often used as indicator of flavor quality of coffee (Dong et al., 2019, Bressanello et al., 2017).

This work set to conduct a detailed sensory evaluation and characterization of key non-volatile and aroma-active compounds that potentially influence flavor difference between these coffee accessions. Quantification of major biochemical non-volatile compounds, including caffeine, chlorogenic acid, trigonelline and sucrose was conducted in green coffee beans using liquid chromatography coupled with mass spectrometry (LC-MS). Head-Space Solid Phase Microextraction-Gas Chromatography was used to determine the profiles of volatile aroma compounds and partial least squares discriminant analysis (PLS-DA) was used to predict discriminant volatiles that could be used to differentiate brews of tested coffee samples. The variation in contents of biochemical compounds in these closely related wild and cultivated accessions could provide further insights into selection for aroma and taste in the local commercial varieties. In addition, the chemistry of coffee taste and aroma generation remain less understood, thus more studies on the relationship between non-volatile compounds and aroma could be useful for predicting new strategies for uncovering the complexity of coffee flavor.

## 2 Materials and methods

### 2.1 Chemicals and reagents

The following chemicals were obtained from Sigma-Aldrich (Munich, Germany): standard caffeine (CAS: 58-08-2), trigonelline (CAS: 6138-41-6), sucrose (CAS: 57-50-1), 5-caffeoylquinic acid (5-CQA) (CAS: 906-33-2) used to represent chlorogenic acid, reference volatile compounds including; 2-furfurylthiol (CAS 98-02-2), hexanal (CAS: 66-25-1), 2-methylfuran (CAS 534-22-5), 2-butanone (CAS: 78-93-3), 2-methyl pyrazine (CAS: 109-08-0), 2,3-dimethyl pyrazine (CAS: 5910-89-4), 2-ethyl-3,5-dimethylpyrazine (CAS:13925-07-0), 2-ethyl-6-methylpyrazine (CAS: 13925-03-6), 2-ethyl-3-methylpyrazine (CAS: 15707-23-0), pyridine (CAS: 110-86-1), 2,3-butanediol (CAS: 513-85-9), benzaldehyde (CAS: 100-52-7), 4-nonanol (CAS: 5932-79-6), phenol (CAS: 108-95-2), 2,3-butanedione (CAS: 207-069-8); HPLC grade water, hexane and basic lead acetate (CAS: 301-04-2).

### 2.2 Sample selection and pre-processing

Details of all the samples used in this study are provided in Table 1. Beans of four commercial varieties and wild Arabica accessions were collected in 2019. For each cultivar, coffee bean samples were collected in triplicates from 15 randomly selected trees, whereas beans of wild accessions were sampled in duplicates from thirty-two trees. Standard pulping, fermentation, washing, sun drying and sorting methods were used during processing (Omondi, 2009). Approximately 500 g of AA grade beans were collected after separation with a 7.2 mm mechanical grading sieve. Moisture balance was used to ensure uniform moisture content of about 10% before storing green samples bean in sealable laminated mylar foil packaging bags to avoid undesired fermentation. The contents of caffeine, chlorogenic acid, trigonelline and sucrose were expressed in dry weight basis (dwb).

**Table 1:** List of coffee accessions used in this study.

Sample ID*	**group**	Sample ID*	**group**	Sample ID*	**group**
SL28-1	Cultivar	SL34-2	Cultivar	MBT-3	Wild
SL28-2	Cultivar	SL34-3	Cultivar	MBT-4	Wild
SL28-3	Cultivar	SL34-4	Cultivar	MBT-5	Wild
SL28-4	Cultivar	SL34-5	Cultivar	MBT-6	Wild
SL28-5	Cultivar	SL34-6	Cultivar	MBT-7	Wild
SL28-6	Cultivar	SL34-7	Cultivar	MBT-8	Wild
SL28-7	Cultivar	SL34-8	Cultivar	MBT-9	Wild
SL28-8	Cultivar	SL34-9	Cultivar	MBT-10	Wild
SL28-9	Cultivar	SL34-10	Cultivar	MBT-11	Wild
SL28-10	Cultivar	SL34-11	Cultivar	MBT-12	Wild
SL28-11	Cultivar	SL34-12	Cultivar	MBT-13	Wild
SL28-12	Cultivar	SL34-13	Cultivar	MBT-14	Wild
SL28-13	Cultivar	SL34-14	Cultivar	MBT-15	Wild
SL28-14	Cultivar	SL34-15	Cultivar	MBT-16	Wild
SL28-15	Cultivar	K7-1	Cultivar	MBT-17	Wild
R11-1	Cultivar	K7-2	Cultivar	MBT-18	Wild
R11-2	Cultivar	K7-3	Cultivar	MBT-19	Wild
R11-3	Cultivar	K7-4	Cultivar	MBT-20	Wild
R11-4	Cultivar	K7-5	Cultivar	MBT-21	Wild
R11-5	Cultivar	K7-6	Cultivar	MBT-22	Wild
R11-6	Cultivar	K7-7	Cultivar	MBT-23	Wild
R11-7	Cultivar	K7-8	Cultivar	MBT-24	Wild
R11-8	Cultivar	K7-9	Cultivar	MBT-25	Wild
R11-9	Cultivar	K7-10	Cultivar	MBT-26	Wild
R11-10	Cultivar	K7-11	Cultivar	MBT-27	Wild
R11-11	Cultivar	K7-12	Cultivar	MBT-28	Wild
R11-12	Cultivar	K7-13	Cultivar	MBT-29	Wild
R11-13	Cultivar	K7-14	Cultivar	MBT-30	Wild
R11-14	Cultivar	K7-15	Cultivar	MBT-31	Wild
R11-15	Cultivar	MBT-1	Wild	MBT-32	Wild
SL34-1	Cultivar	MBT-2	Wild		

*For each cultivar, 15 samples were collected in triplicates, while wild samples were obtained from 32 accessions.

### 2.3 Microwave-assisted extraction and LC-MS analysis

Samples were extracted in triplicate using a previously reported method (Upadhyay et al., 2012) with modifications. Briefly, green beans were ground using pestle and mortar and approximately 5 g of powder sample was dissolved in 20 mL distilled water with an MS-300HS magnetic stirrer (Misung Scientific Co., Ltd., Seoul, Korea) at 200 rpm for 30 min. Hexane (1:6 w/v) was used to defat samples for 1 h in a Soxhlet extraction system. Each sample was extracted with water in triplicate for 5 min at 50 °C and a microwave power of 800 W in a closed system Microwave oven-lab station (Milestone Corporation, Sorisole, Italy), with an inbuilt focused IR sensor and a Magnetron stirrer (SN: 133613; Frequency – 50 Hz). The resulting slurry was cooled and filtered with 0.22 µm filters. A 5 mL aliquot of the extract was bleached with 0.2 mL saturated aqueous basic lead acetate, then diluted with equal volume of HPLC grade prior to LC–MS injection.

An ultra-performance, multiple reaction monitoring liquid chromatography mass spectrometry method with a LC-20ADxR quaternary pump, a CTO-20AC column oven and an SIL-30AC auto injector (Shimadzu, Kyoto, Japan), with a 5.0 μL loop, and interfaced with a LCMS-8040 triple quadrupole mass spectrometer fitted to an electrospray ion source was used following the previously reported methods (Perrone et al., 2008). An EC 125/4 Nucleodur® 100-5C column (125 × 4.0 mm, 5.0 μm, Macherer-Nagel, Germany) was used for chromatographic separations at a constant temperature of 40 °C. Caffeine, trigonelline, sucrose and chlorogenic acid were eluted by a gradient elution in a mobile phase consisting of 0.1% aqueous formic acid (eluent A) and methanol (eluent B), delivered at a flow rate of 0.8 mL/min. Elution was run for 6 min, with a 100:0 ratio of A:B at the beginning, followed by a linear increase to 0:100 at 4 min, held for 1 min, then allowed to decrease to 100:0 in 1 min.

A negative electrospray ionization mode was operated from 0 to 1.5 min to generate formic acid adduct of sucrose, and in the positive mode from 1.5 to 6.0 min to generate caffeine, chlorogenic acid and trigonelline. Desolvation temperature was set at 250 °C, with a nebulizer (N_2_) gas flow at 15 l/min, and mass spectrometer operated in the single ion monitoring (SIM) mode to detect biochemical compounds. Each replicate was randomly injected twice. A column standard injection was done every 5th run to check shift in retention time and mass spectrometer performance throughout experiment. Selected mass-charge transitions were based on retention time and molecular weight of respective standards. LC-MS data processing was carried out using LCMS solution® software (v3.50.346, Shimadzu). Detailed characteristic features of these compounds are shown in Table S1. Quantification of non-volatiles was based on an external six-point curve of pure compounds in the following concentration ranges: caffeine (150 to 4500 mg 100 g^−1^), 5-CQA (122 to 4200 mg 100 g^−1^), trigonelline (72 to 2100 mg 100 g^−1^), sucrose (125–2000 mg 100 g^−1^). Results were statistically assessed by ANOVA and multiple *t*-test for comparison of significant variance between groups at *P* < 0.01. Unsupervised Ward's Hierarchical Cluster Analysis (HCA) was conducted to reveal the Euclidean distances between samples based on LC-MS data set for non-volatile variables of green beans.

## 3 Sensory analysis

### 3.1 Roasting

Approximately 50 g of green coffee beans from ten randomly selected accessions of ‘SL28′, ‘SL34′, ‘K7′, ‘R11′ and wild Arabica were roasted and used for analysis of aroma intensity. Roasting was done to a medium degree at 200 °C for 10 min and to a consistent weight loss of about 20% to allow high accumulation of aroma compounds on a roaster (KN-8828B-2K, Hottop Coffee Roaster, Cranston, RI, USA) as described by Schenker et al. (2002). Roasted samples were then ground at 500 rpm to a fine (approximately < 0.8 mm) powder using a kitchen coffee grinder (Virtuoso+, WA, USA) and immediately packed in air-tight laminate Mylar foil packaging bags and kept for not longer than 2 h prior to GC-analysis to minimize oxidation, diffusion and other effect of storage time on volatile compounds.

### 3.2 Measurement of antioxidant activity and pH in coffee brews

The 2,2-diphenyl-1-picrylhydrazyl (DPPH) radical scavenging assay (Blois, 1958) was used to measure the antioxidant capacity of coffee brew following the method described by Zielinski, Haminiuk, Alberti, Nogueira, Demiate, and Granato (2014). Briefly, 3 mL of methanol extracted coffee brew was mixed with 1 mL of methanolic DPPH solution (1 mM), vortexed and set in the dark for 30 min at room temperature. Subsequently, absorbance was measured using a spectrophotometer (UV–Vis, Agilent-Cary 60) with a wavelength of 517 nm with methanol as a control. A standard curve was prepared using a serial dilution of stock Trolox solution to generate five concentration points (10–100 μmol/L). The absorbance data were used to estimate the antioxidant activity in coffee brews as Trolox equivalent in mmol/L. The percentage radical scavenging activity (%) was estimated using the following equation: [1- (sample abs_517_/control abs_517_)] × 100, where, sample abs_517_ and control abs_517_ represent sample absorbance and control absorbance at 517 nm, respectively. The pH value was measured in 25 mL freshly prepared brews at room temperature using a portable PH60 pH Tester (Apera Instruments, LLC. OH, USA).

### 3.3 Analysis of taste attributes and aroma intensity in coffee brews

Attributes of coffee brew samples were estimated by an experienced panel of five accessors (three males and two females aged 27–41). All judges had a broad experience and training on the Specialty Coffee Association of America's cupping (SCAA) guidelines (Lingle, 2001). Briefly, 10 g of ground coffee powder was mixed with 100 mL hot water in arbitrarily coded glass jars and subjected to evaluation of attributes at 65 °C, including general impression, caramel-like, coffee-like, bitterness, balance, full-body, acidic, fruity notes, nutty and smoky characteristics. Each attribute was scored on a scale of 0 to 10, where 0 represented non perceptible intensity and 10 represented very strong intensity. Results were analyzed with spider graph in excel. In addition, aroma intensity was evaluated on a 10-centimeter unstructured line scale, with ‘no detection’ and ‘strong intensity’ on the left and right, respectively.

### 3.4 Head-space solid phase microextraction and measurement of volatile compounds using gas chromatography (GC)

Approximately 3 g of ground roasted coffee samples was transferred into 20 mL vials. A 1 mL standard solution (43.3 μg of 4-nonanol in 40 mL dichloromethane) was added, and then tightly sealed. The vials were equilibrated in a water bath for 10 min at 60 °C with 400 rpm agitation. To optimize extraction time and temperature that are the key factors influencing quality of volatile extraction, samples were tested by incubation at different temperatures (40, 50, 60 °C) at three-time intervals (7, 23, 40 min), with three replicates for each condition. A DVB/CAR/PDMS StableFlex, 50/30 μm, 1 cm fibre (Supelco, Bellefonte, USA) was exposed to the headspace of samples, and then desorbed into the GC injector port for 5 min at 250 °C in a splitless mode.

Analytes were separated in an Agilent 7890B GC system interfaced with a 5977B-Network-mass selective detector (MSD, Agilent, USA) on a DB-WAX column (30 m × 0.25 mm i.d., 0.5 μm, J&W Scientific, USA). Helium carrier gas was supplied at the rate of 2 mL min^−1^ and the total GC runtime was 70 min. An initial temperature of 50 °C for 4 min was applied, then it was slowly increased to 250 °C at 4 °C/min and held for 5 min, followed by a final hold at 250 °C for 10 min. The mass-spectrometry (MS) was operated in an electron-impact mode at 70 eV. Ion source and quadrupole temperatures were set at 230 °C and 150 °C, respectively. Acquisition in the range of *m*/*z* 35–430 was in full scan mode with a 3 min solvent delay time. Identification was based on retention index, Wiley 6, and installed NIST 98 mass spectral data libraries. Some identified compounds were confirmed by injecting their chemical standards into the GC-MS system. Retention indices of compounds were calculated using *n*-alkane (C_8_–C_30_) series (Sigma-Aldrich, USA). For Quantitative analysis, concentrations of volatile compounds were calculated using an internal standard solution of 4-nonanol in 40 mL dichloromethane at a concentration of 43.3 μg/L. Absolute concentrations of volatile compounds were determined by relating their peak intensities to the intensity of known amount of 4-nonanol in the sample. The concentrations of volatile compounds are expressed in μg/L.

### 3.5 Partial least squares discriminant analysis (PLS-DA)

PLS-DA model was used to find the fundamental relations between coffee accessions (the outcome or response variable) and the normalized spectra data of volatile compounds (predictor variable). MS peak lists data containing a three-column CSV list with sample id, retention time and peaks intensities was imported, and ‘SL28′ was used as a reference sample for data normalization due to its high sensory scores. The Variable Importance in Projection (VIP) score plot was estimated to determine the potential discriminant volatiles contributing to flavor difference among tested coffee accessions in Metaboanalyst software v.5 (Chong et al., 2019), and scores > 1 were considered significant. Permutation test with 1000-fold repetitions was performed to test the accuracy of multivariate models using Fisher’s least significant difference (LSD) at *P* < 0.05. Goodness of fit (*R*^2^) and predictability (*Q*^2^) were used to test the quality of model.

### 3.6 Statistical analysis

Statistical analyses were performed in Prism v.4.03 for Windows (Graph- Pad Software Inc.). Cup scores of sensory analyses were evaluated by two-way ANOVA. Unless stated, analysis of each parameter was determined in triplicate measurement and statistical significance inferred at *P* < 0.05.

## 4 Results and discussion

### 4.1 Difference in non-volatile contents among the main commercial *C. arabica* cultivars in Kenya

**Fig. 1:**
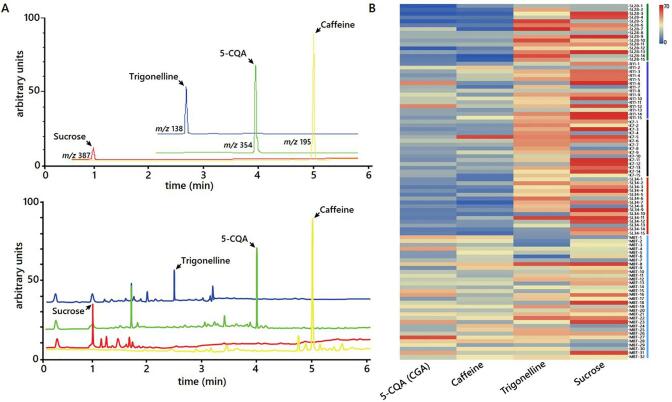
Quantification of biochemical compounds in green coffee beans of different accessions. A, Typical liquid chromatographic separation of reference standards (top), and identification of compounds in sample extract (bottom) with retention time of 4.05, 5.03, 2.55 and 0.98 min for 5-caffeoylquinic acid (5-CQA), caffeine, trigonelline and sucrose, respectively. B, Heat-map showing average concentration of biochemical compounds in three replicate measurements for individual sample tested among coffee accession groups. All data for each sample are means ± SD of triplicate measurements. (For interpretation of the references to color in this figure legend, the reader is referred to the web version of this article.)

**Fig. 2:**
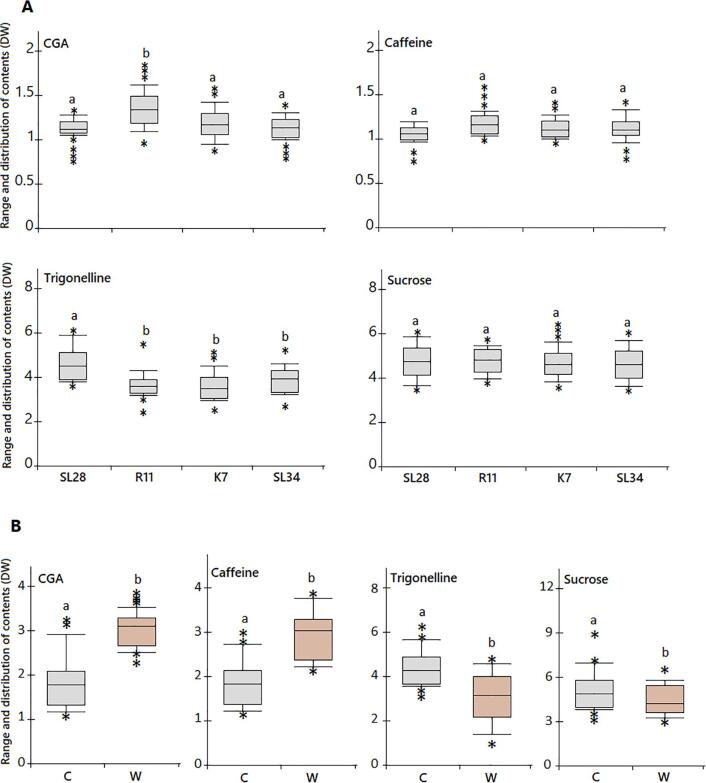
Box plots showing distribution of concentrations for biochemical compounds within *C. arabica* accessions (A) and between wild and cultivated *C. arabica* accessions (B). The horizontal lines within the boxes are the mean values. The box indicates distribution for 50% of the data, with approximately 99% of the data falling within the whiskers. The data outside these whiskers are indicated by asterisk. C, cultivated *C. arabica*; W, wild *C. arabica* accessions. Different lowercase alphabetical letters represent significance difference at *P* < 0.01, *t*-test.

The contents of non-volatile compounds, including caffeine, 5-caffeoylquinic acid (CGA), trigonelline and sucrose, were determined in green beans of four key commercial *C. arabica* cultivars based on the calibration curves of the standards (Fig. 1A). Quantitative analysis revealed a wide variation in the non-volatile contents among fifteen samples from each accession (Fig. 1B). A comparable CGA content was observed in three cultivars, ‘SL28′, ‘K7′ and ‘SL34′, while ‘R11′ had a significantly higher content of 17.0 g/kg DW (*P* < 0.01, Fig. 2A). CGA decompose to form quinic and caffeic acids, which are associated with astringency, bitterness and acidity in beverage and lowering its content is one of the key breeding targets in coffee breeding (Seninde & Chambers, 2020). ‘R11′ is a relatively modern canephoroid genome-introgressed cultivar developed from a series of crosses with several varieties, which could explain its high CGA content and perceived low beverage quality. Similarly, trigonelline was significantly accumulated in ‘SL28′ with 48.5 g/kg DW, while a comparable content was observed in the other three cultivars. Trigonelline, is an alkaloid and one of the key coffee flavor determinants, which is broken down into nicotinic acid, pyrroles and pyridine to produce desirable aroma attributes, such as toasted, nutty and roasted notes, through Maillard reactions during roasting (Farah et al., 2006, Ky et al., 2001). Therefore, the presumed high beverage quality of ‘SL28′ could be partially attributed to its higher trigonelline content.

Sucrose is caramelized in Strecker and Maillard reactions during roasting, generating furans and sulfur compounds with positive flavor properties such as sweet, caramellic, coffee-like aroma and desirable color (Seninde & Chambers, 2020). However, higher sucrose concentration with no significant difference among the tested cultivars was observed. This suggests that sucrose could be less responsible for determining flavor difference among these coffee cultivars. Similarly, caffeine accumulation was comparable among the tested cultivars. Given the fact that caffeine is thermal stable (Vignoli et al., 2014), it is an unlikely component responsible for flavor difference among the tested coffee cultivars. In short, diverse accumulation patterns of non-volatiles were observed in the beans of the four *C. arabica* cultivars used in this study. Similarly, a comparable number of samples were previously used to demonstrate significant variation in biochemical composition and quality differences in beans of five traditional coffee cultivars (Bertrand, Vaast, Alpizar, Etienne, Davrieux, & Charmetant, 2006). CGA and trigonelline seem to be drivers of desired organoleptic coffee qualities, and the variation in their content among *C. arabica* cultivars could be due to artificial selection.

Wide variation in non-volatile contents was also observed among individual samples of each cultivar (Table S2). Variation in CGA content ranged from 1.5 to 2.1 folds, while fold changes, 1.8–3.4, 3.0–5.4, and 2.4–4.6, were observed for caffeine, sucrose, trigonelline contents, respectively. Environmental factors such as altitude and shade environment and post-harvest processing have strong influence on accumulation of non-volatile compounds (Cheng et al., 2016, Sunarharum et al., 2014, Geromel et al., 2008), which could also explain the observed differences in their contents in these coffee varieties. For example, the wet processing technique which is predominantly practiced in Kenya has been shown to enhance loss of CGA during soaking due to its water-soluble property (de Melo Pereira et al., 2015).

Principal component analysis (PCA) based on non-volatile data revealed that cultivated accessions formed a cluster that separated from wild accessions, with PC1 and PC2 explaining a total of 87.1 % of the observed variance (Fig. S1A). This was consistent with the hierarchical clustering patterns (Fig. S1B). These results suggest a potential selection on non-volatile components during the process of coffee domestication.

### 4.2 Comparison of non-volatile contents in green beans between cultivated and wild *C. arabica* accessions

Our previous study showed that the wild *C. arabica* in Marsabit mountain area is genetically closely related to cultivars in Kenya (Ogutu et al., 2016). Thus, we measured non-volatile contents in a collection of 32 wild *C. arabica* accessions. An obvious difference in non-volatile contents was observed in the wild *C. arabica* accession (Fig. 1B), with 4.4-, 2.3-, 4.3-, and 9.4-fold changes in CGA, caffeine, sucrose and trigonelline contents, respectively (Table S2). The distribution in non-volatile contents revealed that caffeine and CGA contents were significantly higher (*P* < 0.01) in wild *C. arabica* accessions than cultivars, while opposite result was observed for trigonelline and sucrose contents (Fig. 2B). This finding is consistent with a previous report (Ky et al., 2001). Increased sucrose and trigonelline contents along with decreased caffeine and CGA contents are associated with high beverage quality (Tran et al., 2018, Richard et al., 2007). Variation in non-volatile contents is affected by coffee variety (Perrois et al., 2015, Vidal et al., 2010, Yapo et al., 2003). Thus, it seems that selection for non-volatile contents has occurred during the domestication of *C. arabica* varieties.

### 4.3 Comparison of antioxidant capacity, pH and sensory attributes in brews between cultivated and wild *C. arabica* accessions

Coffee is a major dietary source of antioxidants with numerous health benefits, and its overall acceptance is mainly determined by bitterness and astringency which are associated with acidity (Seninde & Chambers, 2020). The pH and antioxidant activity of the tested accessions are shown in Table 2. Wild *C. arabica* was more acidic with a pH of 4.7 compared with cultivated accessions with pH ranging from 5.0 to 5.3. Likewise, antioxidant activity was higher in wild *C. arabica* (19.1 Trolox/L) than in cultivated accessions (11.7 – 16.4 Trolox/L). Antioxidant activity was significantly anti-correlated with the pH value (*r* = −0.97, *P* < 0.01). Moreover, significant correlations were also observed between either pH or antioxidant activity and the contents of non-volatile compounds, including trigonelline, CGA and caffeine, but no significant correlation with sucrose (Table S3).

**Table 2:** Analysis of pH and antioxidant activity in roasted coffee.

Measurement	SL28	SL34	K7	R11	Wild
pH	5.3 ± 0.01^a^	5.2 ± 0.01^b^	5.0 ± 0.01^b^	5.0 ± 0.01^b^	4.7 ± 0.02^c^
DPPH (mmol Trolox/L)	11.7 ± 0.21^a^	14.4 ± 0.36^a^	16.4 ± 0.29^b^	16.3 ± 0.11^b^	19.1 ± 0.34^c^

Lowercase letters represent statistical significance at (*P* < 0.05). Results are means of three replicate measurements.

**Fig. 3:**
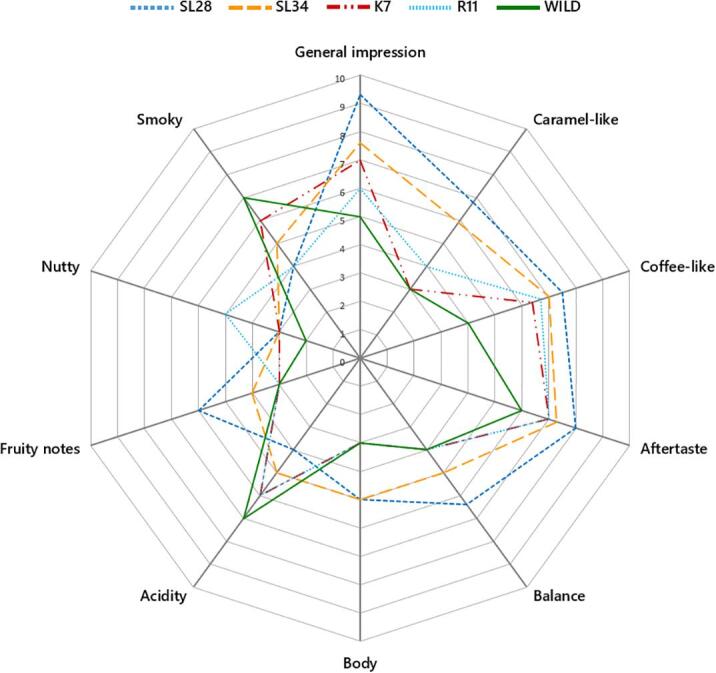
Descriptive sensory analysis of brew attributes of different coffee accessions. Sensory scores are means of three replicate tests.

Sensory attributes evaluated by a panel of five assessors revealed a significant difference in aroma intensity between cultivated and wild accessions (Table S4). Aroma intensity varied significantly among cultivars, with the highest average score of 7.8 in ‘SL28′ and the lowest average score of 7.3 in ‘K7′. Correlation analysis showed that aroma intensity strongly correlated with the trigonelline content, but had relatively weak correlations with the contents of other non-volatile compounds (Table S3). Significant differences in evaluated sensory descriptors were observed between cultivated varieties and wild *C. arabica* accessions, with the highest scores for general impression, caramel-like, aftertaste, balance, and fruity notes attributes in ‘SL28′, while the highest scores for acidity and smoky attributes observed in the wild *C. arabica* was (Fig. 3). Overall, wild *C. arabica* showed inferior sensory scores relative to cultivars. Among cultivars, ‘SL28′ and ‘SL34′ were significantly less acidic and smoky and with higher scores in other sensory attributes than ‘K7′ and ‘R11′, which is consistent with previous finding that SL-varieties have superior brew qualities (Agwanda et al., 2003). Altogether, these results suggested an improved brew quality in cultivated accessions compared to wild *C. arabica* accessions, which could be attributed to changes in accumulation of both non-volatile and volatile compounds (Rao, & Fuller, 2018).

### 4.4 The composition of volatile compounds in cultivated and wild *C. arabica* accessions

To determine the volatile components in roasted coffee using GC-MS analysis, extraction with 40 min exposure at 60 °C that corresponded to the highest total peak areas was used as the optimized condition (Fig. S2). As a result, a total of 141 volatiles were detected, of which, 15 compounds were confirmed using reference standards. The identified compounds belonged to 15 chemical families, with members of pyrazines, volatile phenols, furans, aldehydes, ketones, acids as the most represented volatiles in brews (Table S5). This is consistent with a previous study by (Caporaso et al., 2018). No obvious difference in the number of volatile compounds was observed among tested coffee accessions, with 120, 125, 126, 130 and 119 volatile compounds in ‘SL28′, ‘SL34′, ‘K7′, ‘R11′ and wild *C. arabica* accessions, respectively. Quantitative analysis of identified volatile revealed a wide variation in their total concentrations among the tested accessions, with 34.27, 30.24, 27.82, 28.44, and 23.45 mg/L in ‘SL28′, ‘SL34′, ‘K7′, ‘R11′ and the wild *C. arabica*, respectively. The comparatively lower concentration of aroma compounds observed in wild *C. arabica* is consistent with a previous study (Nijssen et al., 1996).

Higher concentration of volatile compounds in SL-varieties may be one of the key factors contributing to their superior brew quality (Caporaso et al., 2018). Notably, the wild *C. arabica* accumulated a substantial amount of volatile phenolic compounds, which are mostly derived from thermal degradation of chlorogenic acids (Sunarharum et al., 2014). This is plausible due to the above finding that the wild *C. arabica* had the highest CGA content.

### 4.5 Identification of discriminant volatiles among coffee accessions

**Fig. 4:**
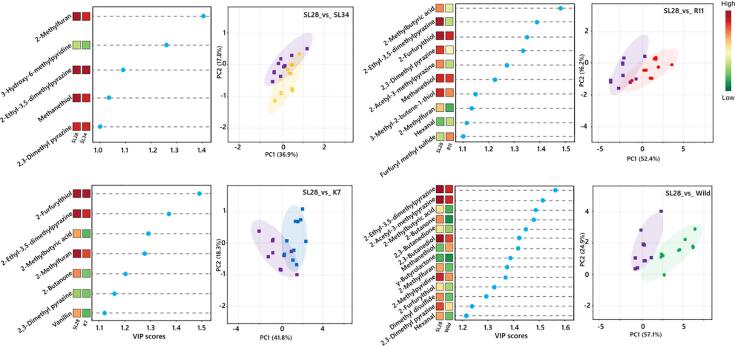
The variable importance in projection scores of candidate volatiles and PCA plots by PLS-DA. Loading plots of potential discriminant volatiles with VIP threshold > 1 and their concentration indicated in colored boxes (left). PCA plots showing the clustering patterns between ‘SL28′ and different coffee accessions based on the discriminant volatiles (right). Ellipses represent 95 % confidence intervals of each coffee accession group.

Since the above analysis indicated that ‘SL28′ had superior coffee brew quality, it was used as a reference accession to screen discriminant volatiles using PLS-DA models. The VIP scores were used to rank compounds according to their significance in defining the observed PCA clustering patterns in the tested models. As a result, 18 discriminant volatiles were ranked with VIP scores > 1 as the most potential compounds that could be used as biomarkers in the sensory evaluation and grading of coffee brews from these varieties (Fig. 4). Of these discriminant volatiles, 5, 7, 10 and 14 were the key odorants contributing to the flavor difference between ‘SL28′ and each of ‘SL34′, ‘K7′, ‘R11′ and wild *C. arabica*, respectively. The 18 discriminant volatiles comprised of 3 pyrazines, 3 thiols, 2 pyridines, 2 ketones, 2 sulfur compounds, and one each for furan, acid, lactone, volatile phenol, aldehyde, and alcohol. Notably, two of the three pyrazines, 2-ethyl-3,5-dimethylpyrazine and 2,3-dimethylpyrazine, were identified in all comparative models. Consistently, thiols such as 2-furfurylthiol and pyrazines, including 2-ethyl-3,6-dimethylpyrazine, 2-ethyl-3,5-dimethylpyrazine, 2,3-diethyl-5-methylpyrazine are key odor-relevant compounds that have previously been shown to generate roasty, earthy, sulfurous and chocolaty aromas which are associated with important freshness perception in coffee brews (Sun et al., 2020, Sunarharum et al., 2014, Poisson et al., 2009, Mayer and Grosch, 2001).

Principal components loading plots revealed that identified volatile compounds could discriminate between ‘SL28′ and wild *C. arabica* (Fig. 4). Comparison of SL28_vs_Wild showed the largest total contribution of PC1 and PC2 in explaining the variance with 82%, followed by comparisons of SL28_vs_R11, SL28_vs_K7 and SL28_vs_SL34 each with 68.6%, 60.1% and 54.7%, respectively. Overall, the concentrations of the discriminant volatile compounds were significantly higher in cultivated accessions than in wild *C. arabica* accessions (Table S5), suggesting that their increased accumulation is crucial for the improved quality of commercial varieties.

## 5 Conclusion

A wide variation in the contents of non-volatile and volatile compounds was observed among the AA high quality grade coffee beans of locally developed commercial varieties in Kenya along with their closely related wild relatives. High levels of both caffeine and CGA accumulation negatively impact coffee flavor, and their decreased accumulation contributes to improved quality in commercial varieties. By contrast, commercial varieties significantly accumulated higher sucrose and trigonelline as well as volatile compounds than wild *C. arabica* accessions. Notably, the trigonelline content is strongly correlated with the main quality traits of coffee brews, such as pH, aroma intensity and antioxidant activity, suggesting the crucial roles of trigonelline in the improvement of commercial varieties in Kenya. Pyrazine- and thiol-type volatiles play important roles in determining coffee flavor and could serve as discriminant compounds for identification of coffee brews.

## CRediT authorship contribution statement

**Collins Ogutu:** Conceptualization, Methodology, Investigation, Writing – original draft. **Sylvia Cherono:** Data curation, Writing – review & editing. **Charmaine Ntini:** Data curation, Writing – review & editing. **Lu Wang:** Software, Validation. **Yuepeng Han:** Funding acquisition, Project administration, Writing – review & editing.

## Declaration of Competing Interest

The authors declare that they have no known competing financial interests or personal relationships that could have appeared to influence the work reported in this paper.
