# Supplementary material for: Comprehensive analysis of quality characteristics in main commercial coffee varieties and wild Arabica in Kenya
Source: Food Chem X. 2022 Mar 25;14:100294. doi: 10.1016/j.fochx.2022.100294 (PMC8958313; doi:10.1016/j.fochx.2022.100294)
Supplement: Supplementary data 1 [file mmc1.docx]

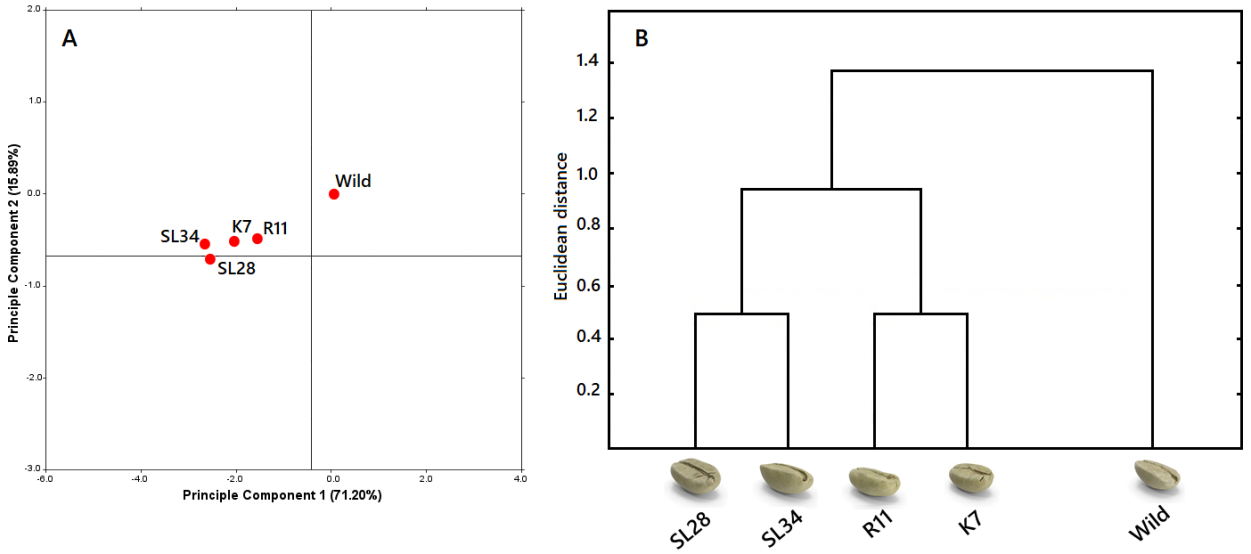


Fig. S1. The principal component analysis (A), and the dendrogram (B) showing differentiation based on the four non-volatile matrix in green beans of coffee accessions.


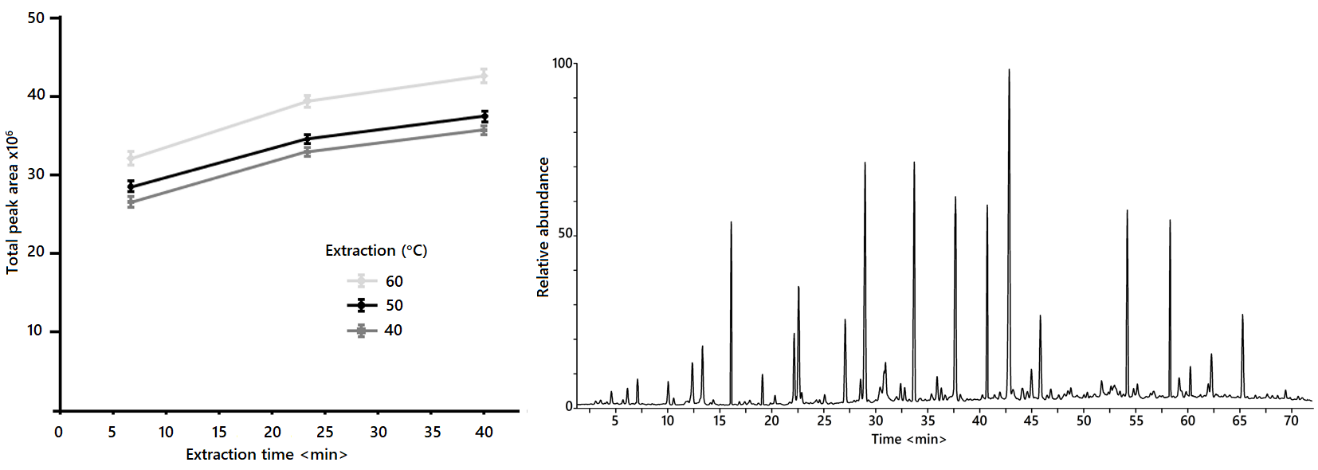


Figure S2. HS-SPME optimization and GC- MS analysis. HS-SPME parameters showing optimized conditions of 40 min extraction period at 60 °C after a 10 min 50/30 μm DVB/CAR/PDMS exposure, (Left). Total peak area was analyzed as a function of extraction temperature. A typical total ion chromatogram (TIC) obtained from roasted coffee sample by HS SPME GC-MS analysis, (Right).

Table S1. The LC MS/MS characterization of the four non-volatile compounds in coffee samples

| Compound | *t*R (min) | Formular | Parent ion m/z | MS/MS ions |
| --- | --- | --- | --- | --- |
| Caffeine | 5.03 | C_8_H_10_N_4_O_2_ | 195 | 195, 163, 151 |
| Trigonelline | 2.55 | C_7_H_7_NO_2_ | 138 | 138, 98, 74 |
| CGA (5-CQA) | 4.05 | C_16_H_18_O_9_ | 354 | 354, 193, 182, 175, 163 |
| Sucrose | 0.98 | C_12_H_22_O_11_ | 387 | 387, 364, 273, 246 |

Identification was based on comparison with both chemical standards and previously reported MS fragments. *t*R, retention time.

Table S2. Comparison of non-volatile contents in green beans of tested coffee accessions (g/kg DW)

| Acc. | | CGA | | | |  | | Caffeine | | |  | | Sucrose | | | |  | | Trigonelline | | | |
| --- | --- | --- | --- | --- | --- | --- | --- | --- | --- | --- | --- | --- | --- | --- | --- | --- | --- | --- | --- | --- | --- | --- |
|  |  | Min | Max | | Av. |  | | Min | Max | Av. |  | | Min | Max | | Av. |  | | Min | Max | Av. |  |
| SL28 | | 8.63 | 18.01 | 12.44 | | | 6.67 | | 12.09 | 11.37 | | 22.80 | | | 69.21 | 47.31 | | 24.24 | | 69.70 | 48.49 |  |
| R11 | | 12.06 | 21.03 | 17.02 | | | 8.76 | | 25.41 | 13.50 | | 13.55 | | | 69.54 | 48.11 | | 18.42 | | 53.10 | 38.25 |  |
| K7 | | 13.61 | 20.18 | 13.40 | | | 8.78 | | 17.23 | 12.51 | | 13.91 | | | 68.96 | 46.57 | | 22.49 | | 54.58 | 38.13 |  |
| SL34 | | 9.98 | 19.57 | 12.93 | | | 3.72 | | 12.68 | 12.08 | | 12.77 | | | 69.12 | 46.78 | | 12.47 | | 57.41 | 43.28 |  |
| Wild | | 14.52 | 63.97 | 25.59 | | | 19.07 | | 43.43 | 17.02 | | 15.67 | | | 66.98 | 42.24 | | 6.70 | | 63.02 | 24.13 |  |

Min and Max values are means ± SD of triplicate measurements, *P* < 0.01, *t*-test.

Table S3. Correlation between the contents of non-volatile compounds and coffee brew quality

| Trait | CGA | Caffeine | Sucrose | Trigonelline |
| --- | --- | --- | --- | --- |
| pH | -0.91* | -0.95** | 0.78 | 0.99** |
| Antioxidant activity | 0.82* | 0.88* | -0.69 | -0.96** |
| Aroma intensity | -0.79 | -0.86* | 0.69 | 0.95** |

* *P* < 0.05, ** *P* < 0.01.

Table S4. Assessment of aroma intensity scores in coffee brews

| Accessor | SL28 | SL34 | K7 | R11 | Wild |
| --- | --- | --- | --- | --- | --- |
| 1 | 7.5 | 7.4 | 7.5 | 7.8 | 7.3 |
| 2 | 8.3 | 7.9 | 7.2 | 7.1 | 7.0 |
| 3 | 7.7 | 7.5 | 7.2 | 7.3 | 7.1 |
| 4 | 8.0 | 7.8 | 7.4 | 7.3 | 7.2 |
| 5 | 7.6 | 7.4 | 7.1 | 7.4 | 7.0 |
| Average | 7.8^a^ | 7.6^b^ | 7.3^c^ | 7.4^c^ | 7.1^d^ |

The scores are means of three replicate tests. Lowercase letters represent statistical significance at *P* < 0.05.

Table S5. Distribution of volatile compounds and variation in their concentration in brews of tested coffee accessions

| **Compounds** | **LRI^a^** | **ID^b^** | **Concentration (μg/L), n=10** | | | | |
| --- | --- | --- | --- | --- | --- | --- | --- |
|  |  |  | **SL28** | **SL34** | **K7** | **R11** | **Wild** |
| **Pyrazines:** |  |  |  |  |  |  |  |
| 2-Metoxy-3-(2methylpropyl) pyrazine | 1223 | LRI/MS | 136 ± 2.14 | 134 ± 0.24 | 138 ± 1.21 | 126 ± 2.34 | 122 ± 3.18 |
| 3-Ethyl-2,5-dimethylpyrazine | 1503 | LRI/MS | 387 ± 3.51 | 316 ± 1.33 | 303 ± 0.14 | 312 ± 4.11 | 196 ± 1.64 |
| 2-Methyl pyrazine | 1228 | LRI/MS/IS | 68 ± 5.63 | 56 ± 4.19 | 51 ± 3.72 | 54 ± 5.13 | 46 ± 4.19 |
| 2-Propylpyrazine | 1430 | LRI/MS | 281 ± 1.11 | 124 ± 7.17 | 81 ± 6.55 | 79 ± 1.48 | nd |
| 2-Ethyl-5-methylpyrazine | 1392 | LRI/MS | 416 ± 3.88 | 412 ± 2.15 | 413 ± 3.84 | 418 ± 6.29 | 358 ± 7.54 |
| 2-Acetyl-3-methylpyrazine | 1699 | LRI/MS | 713 ± 8.22 | 748 ± 2.81 | 316 ± 6.12 | 211 ± 3.15 | 187 ± 4.15 |
| 1-(6-methyl-2-pyrazinyl)-1-ethanone | 1697 | LRI/MS | 59 ± 5.19 | 51 ± 4.54 | 89 ± 5.64 | 108 ± 5.04 | 133 ± 2.61 |
| 2-Acetyl pyrazine | 1627 | LRI/MS | 611 ± 5.54 | 431 ± 1.76 | 396 ± 0.94 | 394 ± 3.17 | 329 ± 5.19 |
| 2-Methyl-5-propenyl-(E)-pyrazine | 1793 | LRI/MS | 319 ± 4.66 | 318 ± 3.61 | 211 ± 3.86 | 203 ± 5.81 | 201 ± 6.03 |
| 2,3-Dimethyl pyrazine | 1344 | LRI/MS/IS | 1082 ± 1.31 | 867 ± 4.18 | 499 ± 7.14 | 489 ± 3.96 | 466 ± 9.18 |
| 2,6-Dimethyl pyrazine | 1326 | LRI/MS | 96 ± 2.18 | 113 ± 1.23 | 142 ± 3.11 | 141 ± 1.31 | 108 ± 2.66 |
| 2,3,5-Trimethyl pyrazine | 1427 | LRI/MS | 22 ± 8.33 | 20 ± 3.41 | 17 ± 2.56 | 12 ± 5.44 | 18 ± 7.12 |
| 2,5-Diethylpyrazine | 1439 | LRI/MS | 1085± 1.16 | 1009 ± 2.17 | 832 ± 5.43 | 812 ± 3.19 | 811 ± 5.315 |
| 2-Methyl-5-vinylpyrazine | 1496 | LRI/MS | 32 ± 1.51 | 29 ± 2.11 | 18 ± 2.13 | nd | 10 ± 1.12 |
| 2-Ethyl-3,5-dimethylpyrazine | 1450 | LRI/MS/IS | 1421 ± 1.18 | 1312 ± 4.21 | 1102 ± 1.91 | 1072 ± 2.18 | 1012 ± 2.31 |
| 2-Ethyl pyrazine | 1384 | LRI/MS | 222 ± 3.41 | 194 ± 2.66 | 132 ± 6.11 | 134 ± 5.16 | 119 ± 5.49 |
| 2-Ethyl-6-methylpyrazine | 1390 | LRI/MS/IS | 142 ± 1.62 | 139 ± 3.18 | 176 ± 5.19 | 182 ± 3.12 | 212 ± 3.54 |
| 2-Ethyl-3-methylpyrazine | 1410 | LRI/MS/IS | 912 ± 5.19 | 897 ± 2.44 | 842 ± 7.51 | 852 ± 2.81 | 132 ± 6.15 |
| 5H-5-(Methyl)-6,7-dihydro cyclopentapyrazine | 1631 | LRI/MS | 14 ± 7.17 | 42 ± 4.15 | 52 ± 7.18 | 128 ± 5.13 | 122 ± 6.38 |
| 2-Ethyl-3-methyl pyrazine | 1467 | LRI/MS | 442 ± 9.11 | 386 ± 2.81 | 346 ± 6.19 | 333 ± 4.26 | 326 ± 4.87 |
| 2-isobutyl-3-methoxypyrazine | 1170 | MS/MS | 84 ± 2.81 | 71 ± 8.57 | nd | 98 ± 4.73 | nd |
| **TOTAL** |  |  | **8521** | **7672** | **6156** | **6158** | **4908** |
| **Pyridines**: |  |  |  |  |  |  |  |
| 2-Methyl pyridine | 1239 | LRI/MS | 214 ± 1.61 | 166 ± 2.13 | 158 ± 5.33 | 152 ± 3.14 | 139 ± 8.14 |
| 3-Ethyl pyridine | 1355 | LRI/MS | 188 ± 2.81 | 199 ± 0.41 | 184 ± 4.17 | 191 ± 1.71 | 201 ± 2.18 |
| Pyridine | 1180 | LRI/MS/IS | 576 ± 5.44 | 436 ± 6.17 | 416 ± 4.56 | 429 ± 2.82 | 371 ± 3.75 |
| 3-Methyl pyridine | 1310 | LRI/MS | 232 ± 5.33 | 224 ± 2.16 | 192 ± 6.31 | 175 ± 5.88 | 169 ± 5.71 |
| 3-hydroxy-6-methylpyridine | 2433 | LRI/MS | 447 ± 6.72 | 219 ± 8.12 | 107 ± 7.47 | 98 ± 8.49 | 102 ± 0.49 |
| 3-pyridinol | 2451 | LRI/MS | 102 ± 1.41 | 106 ± 6.39 | 316 ± 1.87 | 322 ± 3.93 | 242 ± 3.51 |
| **TOTAL** |  |  | **1759** | **1350** | **1373** | **1367** | **1224** |
| **Alcohols:** |  |  |  |  |  |  |  |
| 5-indanol | 1532 | LRI/MS | 137 ± 5.08 | 182 ± 2.39 | 357 ± 6.37 | 392 ± 1.31 | 142 ± 7.55 |
| 2-furanmethanol | 2120 | LRI/MS | 392 ± 6.31 | 112 ± 1.39 | 100 ± 8.68 | 110 ± 2.39 | 72 ± 5.15 |
| 2-Phenoxyethanol | 1874 | LRI/MS | nd | 11 ± 9.18 | 14 ± 3.91 | 12 ± 3.87 | 16 ± 9.38 |
| Benzyl alcohol | 1911 | LRI/MS | 92 ± 5.66 | 81 ± 4.14 | 102 ± 1.19 | 99 ± 8.37 | 164 ± 4.37 |
| Phenylethyl alcohol | 1661 | LRI/MS | 319 ± 6.34 | 312 ± 2.37 | 42 ± 6.37 | 162 ± 1.41 | 72 ± 1.33 |
| Furfuryl alcohol | 1581 | LRI/MS | 292 ± 2.34 | 288 ± 6.31 | 236 ± 3.11 | 212 ± 8.44 | 104 ± 3.91 |
| 2,3-butanediol | 1576 | LRI/MS/IS | 212 ± 1.94 | 241 ± 2.35 | 252 ± 4.42 | 358 ± 5.48 | 686 ± 5.55 |
| 1,3-butanediol | 1617 | LRI/MS | 18 ± 0.25 | 23 ± 1.47 | 31 ± 2.18 | 44 ± 1.16 | 101 ± 3.11 |
| 2-(2-Butoxyethoxy) ethanol | 1820 | LRI/MS | 110 ± 1.16 | 292 ± 3.44 | 480 ± 4.22 | 442 ± 3.88 | 392 ± 6.99 |
| **TOTAL** |  |  | **1572** | **1542** | **1614** | **1831** | **1749** |
| **Aldehydes:** |  |  |  |  |  |  |  |
| Hexanal | 1022 | LRI/MS/IS | 292 ± 4.31 | 311 ± 3.78 | 399 ± 7.87 | 403 ± 1.47 | 512 ± 4.18 |
| Benzaldehyde | 1540 | LRI/MS/IS | 42 ± 3.28 | 57 ± 0.22 | 48 ± 3.48 | 54 ± 0.33 | nd |
| 3-methyl-1,2-butenal | 1394 | LRI/MS | nd | nd | nd | 22 ± 5.15 | 44 ± 3.88 |
| Furfural | 1493 | LRI/MS | 239 ± 1.12 | 221 ± 7.14 | 274 ± 8.24 | 289 ± 2.71 | 244 ± 8.44 |
| 5-methylfurfural | 1618 | LRI/MS | 406 ± 7.33 | 410 ± 0.22 | 414 ± 4.29 | 416 ± 1.02 | 411 ± 5.52 |
| Acetaldehyde | 1374 | LRI/MS | 480 ± 4.22 | 492 ± 1.31 | 399 ± 3.37 | 382 ± 6.31 | 368 ± 9.34 |
| Methylpropanal | 833 | LRI/MS | 221 ± 2.45 | 164 ± 2.77 | 143 ± 9.01 | 317 ± 2.11 | 318 ± 1.87 |
| 2-methylbutanal | 1424 | LRI/MS | 334 ± 4.34 | 284 ± 7.12 | 244 ± 4.35 | 184 ± 4.71 | 189 ± 2.55 |
| 3-methylbutanal | 921 | LRI/MS | 154 ± 1.39 | 148 ± 4.37 | 114 ± 5.07 | 101 ± 1.22 | 91 ± 7.07 |
| 2-methyl-2-pentanal | 820 | LRI/MS | 21 ± 2.18 | 19 ± 2.01 | 17 ± 8.44 | 31 ± 3.71 | 188 ± 2.18 |
| 3-ethyl-benzaldehyde | 1170 | LRI/MS | 111 ± 0.23 | 114 ± 2.58 | 141 ± 9.03 | 152 ± 0.18 | 141 ± 6.43 |
| 2-hydroxy-benzaldehyde | 1048 | LRI/MS | 58 ± 3.26 | 42 ± 4.33 | 36 ± 0.18 | 19 ± 7.61 | 11 ± 1.17 |
| **TOTAL** |  |  | **2358** | **2262** | **2240** | **2370** | **2561** |
| **Thiols:** |  |  |  |  |  |  |  |
| 3-Methyl-2-butene-1-thiol | 1122 | LRI/MS | 892 ± 1.51 | 681 ± 3.44 | 629 ± 8.38 | 617 ± 5.35 | 379 ± 8.89 |
| Methanethiol | 688 | LRI/MS/IS | 1180 ± 1.37 | 1151 ± 6.83 | 1023 ± 9.02 | 1088 ± 1.81 | 977 ± 3.34 |
| 2-furfurylthiol | 1425 | LRI/MS/IS | 1374 ± 8.63 | 1371 ± 4.15 | 1097 ± 5.36 | 1070 ± 6.87 | 940 ± 4.79 |
| 2-Methyl-3-furanthiol | 1310 | LRI/MS | 248 ± 3.71 | 232 ± 5.88 | 218 ± 8.71 | 221 ± 4.84 | 92 ± 8.87 |
| 3-mercapto-3-methyl-1-butanol | 1656 | LRI/MS | 82 ± 7.69 | 92 ± 5.37 | 62 ± 7.37 | 58 ± 5.78 | nd |
| 3-Mercapto-3-methyl-1-butyl formate | 1524 | LRI/MS | 873 ± 3.31 | 974 ± 4.17 | 1072 ± 5.71 | 1086 ± 9.18 | 485 ± 7.48 |
| **TOTAL** |  |  | **4649** | **4501** | **4101** | **4140** | **2873** |
| **Sulfur compounds:** |  |  |  |  |  |  |  |
| 2-methylthiophene | 1092 | LRI/MS | 244 ± 1.91 | 298 ± 5.82 | 448 ± 3.71 | 811 ± 1.91 | 798 ± 9.86 |
| Furfuryl methyl disulfide | 1936 | LRI/MS | 1048 ± 8.01 | 1011 ± 4.81 | 1007 ± 4.82 | 996 ± 9.77 | 518 ± 7.21 |
| Dimethyl disulfide | 1088 | LRI/MS | 898 ± 5.99 | 648 ± 3.78 | 428 ± 3.71 | 248 ± 1.61 | 192 ± 4.67 |
| Furfuryl methyl sulfide | 1477 | LRI/MS | 387 ± 3.68 | 398 ± 3.83 | 578 ± 4.67 | 369 ± 6.55 | 347 ± 6.59 |
| 1,3-thiazole | 1272 | LRI/MS | 79 ± 5.44 | 82 ± 6.37 | 78 ± 3.66 | 32 ± 5.37 | nd |
| **TOTAL** |  |  | **2656** | **2437** | **2539** | **2456** | **1855** |
| **Furans:** |  |  |  |  |  |  |  |
| 2-Furanmethanol | 1651 | LRI/MS | 369 ± 1.11 | 459 ± 1.27 | 488 ± 3.21 | 459 ± 2.44 | 436 ± 0.18 |
| 5-Methyl-2-furfural | 1576 | LRI/MS | 39 ± 1.75 | 36 ± 1.21 | 27 ± 1.54 | 31 ± 1.05 | 231 ± 1.15 |
| Furfural | 1469 | LRI/MS | 284 ± 0.99 | 273 ± 1.88 | 269 ± 3.11 | 266 ± 5.12 | 458 ± 7.18 |
| 2-Acetylfuran | 1510 | LRI/MS | 13 ± 8.43 | 19 ± 5.16 | 18 ± 5.44 | 22 ± 3.18 | nd |
| 5-Furfural | 2469 | LRI/MS | 66 ± 3.41 | 89 ± 8.07 | 78 ± 3.22 | 69 ± 5.12 | nd |
| 2-Methylfuran | 843 | LRI/MS/IS | 1177 ± 5.44 | 944 ± 9.32 | 611 ± 3.44 | 638 ± 1.44 | 549 ± 2.65 |
| 3-Methylfuran | 877 | LRI/MS | 434 ± 1.22 | 451 ± 4.13 | 412 ± 0.78 | 414 ± 2.61 | 76 ± 1.44 |
| 2-Acetyl-5-methylfuran | 1850 | LRI/MS | nd | nd | nd | 18 ± 3.04 | 146 ± 8.22 |
| 2-Acetylfuran | 1488 | LRI/MS | 209 ± 1.07 | 211 ± 3.15 | 219 ± 1.49 | 251 ± 4.81 | 437 ± 0.54 |
| Furfuryl formate | 1511 | LRI/MS | 31 ± 2.38 | 41 ± 0.48 | 33 ± 8.12 | 39 ± 1.31 | nd |
| Furfuryl acetate | 1527 | LRI/MS | nd | nd | nd | 49 ± 3.01 | 163 ± 3.44 |
| 2-butylfuran | 1504 | LRI/MS | 448 ± 1.26 | 418 ± 2.39 | 389 ± 2.71 | 284 ± 6.27 | 179 ± 3.88 |
| Furfuryl alcohol | 1658 | LRI/MS | 71 ± 2.22 | 78 ± 4.25 | 79 ± 5.44 | nd | 200 ± 2.11 |
| **TOTAL** |  |  | **3141** | **3019** | **2623** | **2540** | **2938** |
| **Furanones:** |  |  |  |  |  |  |  |
| Dihydro-2-methyl-3(2H)-furanone | 1280 | LRI/MS | nd | 24 ± 1.11 | 36 ± 3.22 | 41 ± 4.39 | 56 ± 1.01 |
| 2-Methyltetrahydrofuran-3-one | 1284 | LRI/MS | 133 ± 0.17 | 130 ± 2.16 | 127 ± 4.17 | 50 ± 3.28 | nd |
| 5-Methyl-2(3H)-furanone | 1430 | LRI/MS | nd | nd | 28 ± 0.44 | 41 ± 2.11 | 45 ± 8.12 |
| 2(5H)-furanone | 1792 | LRI/MS | 325 ± 0.34 | 422 ± 8.44 | 418 ± 7.21 | 479 ± 2.24 | 418 ± 1.55 |
| **TOTAL** |  |  | **458** | **576** | **609** | **611** | **580** |
| **Lactones:** |  |  |  |  |  |  |  |
| γ-butyrolactone | 1630 | LRI/MS | 188 ± 2.33 | 169 ± 0.14 | 112 ± 7.04 | 125 ± 2.27 | 86 ± 8.19 |
| 4-ethoxycarbonyl-γ-butanolactone | 2502 | LRI/MS | 31 ± 3.01 | 30 ± 5.66 | nd | nd | nd |
| Maple lactone | 1835 | LRI/MS | 227 ± 0.94 | 188 ± 3.22 | 127 ± 1.51 | 124 ± 6.13 | 79 ± 3.57 |
| **TOTAL** |  |  | **446** | **387** | **239** | **249** | **165** |
| **Esters:** |  |  |  |  |  |  |  |
| Ethyl-3-methylbutyrate | 929 | LRI/MS | 330 ± 3.16 | 341 ± 0.18 | 322 ± 1.23 | 300 ± 4.27 | 290 ± 6.08 |
| Furfuryl acetate | 1508 | LRI/MS | 41 ± 1.91 | 48 ± 0.17 | 53 ± 6.11 | 61 ± 1.27 | nd |
| Furfuryl propanoate | 1430 | LRI/MS | 73 ± 0.06 | 62 ± 1.84 | 80 ± 7.13 | 98 ± 4.12 | 60 ± 2.81 |
| Hexyl butyrate | 1559 | LRI/MS | 215 ± 2.47 | 218 ± 4.23 | 189 ± 1.36 | 179 ± 0.19 | 147 ± 4.22 |
| Furfuryl formate | 1791 | LRI/MS | 280 ± 4.91 | 132 ± 3.71 | 78 ± 3.88 | 81 ± 8.11 | 30 ± 1.39 |
| Methyl salicylate | 1547 | LRI/MS | 423 ± 1.18 | 119 ± 0.16 | 125 ± 0.10 | 122 ± 0.88 | 81 ± 1.42 |
| **TOTAL** |  |  | **1362** | **920** | **847** | **841** | **608** |
| **Volatile phenols:** |  |  |  |  |  |  |  |
| 2-methylphenol | 1988 | LRI/MS | 93 ± 2.35 | 102 ± 1.11 | 312 ± 1.87 | 352 ± 0.18 | 332 ± 3.85 |
| 3-methylphenol | 1169 | LRI/MS | 52 ± 4.21 | 58 ± 2.29 | 61 ± 3.71 | 73 ± 1.98 | 112 ± 1.23 |
| Phenol | 1854 | LRI/MS/IS | 38 ± 1.42 | 71 ± 1.83 | 60 ± 0.63 | 55 ± 4.11 | 72 ± 1.91 |
| 2-Acetyl phenol | 1905 | LRI/MS | nd | nd | 88 ± 1.44 | 101 ± 2.37 | 46 ± 1.35 |
| Guaiacol | 2002 | LRI/MS | 302 ± 5.57 | 322 ± 1.23 | 344 ± 3.44 | 233 ± 6.18 | 285 ± 1.23 |
| *p*-Ethyl guaiacol | 2032 | LRI/MS | nd | 37 ± 1.77 | 32 ± 1.61 | 18 ± 1.14 | 89 ± 1.72 |
| 4-Vinylguaiacol | 2182 | LRI/MS | 122 ± 2.71 | 83 ± 1.18 | 75 ± 1.42 | 144 ± 1.38 | 68 ± 4.17 |
| 4-Vinylphenol | 2502 | LRI/MS | 126 ± 0.24 | 112 ± 1.75 | 99 ± 2.73 | 116 ± 5.11 | 155 ± 3.44 |
| 4-Ethylphenol | 2302 | LRI/MS | 45 ± 2.27 | 40 ± 0.88 | 61 ± 3.76 | 60 ± 0.68 | 31 ± 3.15 |
| Vanillin | 2555 | LRI/MS | 427 ± 5.14 | 282 ± 1.55 | 277 ± 1.23 | 281 ± 1.82 | 255 ± 0.27 |
| 4-aminophenol | 2399 | LRI/MS | nd | nd | 28 ± 1.57 | 41 ± 1.22 | 48 ± 1.31 |
| 4-nonylphenol | 2658 | LRI/MS | 133 ± 1.11 | 129 ± 1.71 | 112 ± 4.22 | 110 ± 2.21 | 312 ± 3.66 |
| 4-methylphenol | 2086 | LRI/MS | 214 ± 4.42 | 180 ± 1.52 | 131 ± 1.26 | 82 ± 1.73 | 56 ± 1.45 |
| 2-allyl-5-nitrophenol | 2068 | LRI/MS | 62 ± 1.91 | 52 ± 4.22 | 18 ± 4.31 | 15 ± 0.88 | 22 ± 1.83 |
| **TOTAL** |  |  | **1614** | **1468** | **1698** | **1681** | **1883** |
| **Ketones:** |  |  |  |  |  |  |  |
| 1-Hydroxy-2-butanone | 1381 | LRI/MS | 446 ± 2.17 | 223 ± 1.18 | 151 ± 6.01 | 122 ± 2.33 | 61 ± 1.01 |
| 3-Hydroxy-3-methyl-2-butanone | 1250 | LRI/MS | 61 ± 4.18 | 24 ± 1.02 | 113 ± 1.22 | 140 ± 3.11 | 103 ± 1.18 |
| 1-Hydroxy-2-propanone | 1310 | LRI/MS | 221 ± 1.42 | 233 ± 0.13 | 181 ± 6.07 | 154 ± 1.14 | 162 ± 3.14 |
| 2-Methyl-2-pentanone | 1695 | LRI/MS | 23 ± 1.19 | 16 ± 4.01 | nd | nd | nd |
| 2,3-Butanedione | 985 | LRI/MS/IS | 514 ± 2.29 | 418 ± 2.21 | 409 ± 7.13 | 394 ± 1.33 | 214 ± 4.19 |
| 2-Hydroxy-3-methyl-2-cyclopenten-1-one | 1810 | LRI/MS | 57 ± 4.21 | 44 ± 4.29 | 19 ± 1.13 | 31 ± 4.15 | 14 ± 1.21 |
| 1-(Acetyloxy)-2-propanone | 1478 | LRI/MS | nd | nd | 32 ± 1.22 | 61 ± 1.24 | 50 ± 4.02 |
| 2,3-Pentanedione | 1075 | LRI/MS | nd | 22 ± 0.18 | 16 ± 0.41 | 34 ± 2.47 | 28 ± 2.25 |
| 1-Hydroxy-2-pentanone | 1455 | LRI/MS | 114 ± 0.21 | 81 ± 2.01 | 21 ± 1.29 | nd | 17 ± 1.81 |
| 3-Hydroxy-2-butanone | 1307 | LRI/MS | 48 ± 2.37 | 14 ± 7.29 | nd | 19 ± 3.27 | 12 ± 2.16 |
| 2-Methyl-3-pentanone | 1539 | LRI/MS | 214 ± 5.11 | 203 ± 3.28 | 208 ± 1.34 | 193 ± 4.15 | 179 ± 6.93 |
| 2-butanone | 906 | LRI/MS/IS | 788 ± 1.33 | 215 ± 1.33 | 182 ± 3.28 | 144 ± 4.66 | 105 ± 0.28 |
| **TOTAL** |  |  | **2486** | **1493** | **1332** | **1321** | **945** |
| **Pyrroles:** |  |  |  |  |  |  |  |
| Pyrrole-2-carboxaldehyde | 1148 | LRI/MS | 301 ± 1.22 | 319 ± 1.16 | 322 ± 1.44 | 299 ± 4.18 | 283 ± 2.17 |
| 1H-Pyrrole | 1546 | LRI/MS | 426 ± 3.14 | 422 ± 0.19 | 275 ± 2.13 | 287 ± 9.17 | 195 ± 1.33 |
| 1H-Pyrrole-2-carboxaldehyde | 2031 | LRI/MS | 39 ± 5.13 | 55 ± 1.17 | 47 ± 2.47 | 37 ± 4.98 | 41 ± 1.38 |
| Indole | 2462 | LRI/MS | 62± 0.37 | 59 ± 1.48 | 48 ± 6.11 | 27 ± 7.31 | 13 ± 8.91 |
| 1-furfuryl-2-formylpyrrole | 2241 | LRI/MS | 23 ± 0.44 | nd | 11 ± 1.18 | 14 ± 4.16 | nd |
| 2-acetylpyrrole | 1958 | LRI/MS | 44 ± 5.01 | 56 ± 1.73 | 47 ± 8.13 | 61 ± 1.10 | 50 ± 2.12 |
| 2-formyl-1-methylpyrrole | 1628 | LRI/MS | 512 ± 3.11 | 519 ± 1.42 | 487 ± 3.62 | 419 ± 1.08 | 348 ± 6.13 |
| 4-methylpyrrolo[1,2-a] pyrazine | 2034 | LRI/MS | 36 ± 1.37 | 39 ± 4.22 | 38 ± 4.05 | 42 ± 8.13 | 35 ± 1.58 |
| 1-Methyl-1H-pyrrole | 2008 | LRI/MS | 78 ± 4.03 | 66 ± 1.84 | 67 ± 5.78 | 33 ± 1.62 | nd |
| **TOTAL** |  |  | **1521** | **1535** | **1342** | **1219** | **974** |
| **Acids:** |  |  |  |  |  |  |  |
| Propanoic acid | 1551 | LRI/MS | 85 ± 5.14 | 86 ± 2.11 | 91 ± 3.13 | 99 ± 7.51 | 89 ± 4.22 |
| Salicylic acid | 1294 | LRI/MS | nd | nd | 18 ± 2.44 | 14 ± 3.17 | 27 ± 2.11 |
| Hexanoic acid | 1848 | LRI/MS | nd | 16 ± 1.11 | 22 ± 0.16 | 19 ± 5.21 | 36 ± 1.93 |
| Acetic acid | 1464 | LRI/MS | 31 ± 1.19 | 38 ± 0.18 | 46 ± 2.17 | 44 ± 3.13 | 51 ± 2.82 |
| Pentanoic acid | 1647 | LRI/MS | 104 ± 0.15 | 111 ± 2.33 | 125 ± 0.13 | 142 ± 1.18 | 217 ± 1.27 |
| 2-/3-Methylbutanoic acid | 1690 | LRI/MS | nd | nd | nd | 32 ± 3.11 | 24 ± 5.12 |
| Isovaleric acid | 1692 | LRI/MS | 23 ± 3.12 | 16 ± 0.14 | nd | 39 ± 2.16 | 29 ± 5.32 |
| Nonanoic acid | 2148 | LRI/MS | 215 ± 0.23 | 266 ± 2.51 | 352 ± 1.14 | 341 ± 2.33 | 275 ± 4.61 |
| 2-methylbutyric acid | 1649 | LRI/MS | 518 ± 1.30 | 128 ± 2.27 | 311 ± 1.83 | 355 ± 3.17 | 287 ± 1.38 |
| 3-methylbutyric acid | 1705 | LRI/MS | nd | nd | 12 ± 0.02 | 25 ± 0.03 | 10 ± 0.12 |
| **TOTAL** |  |  | **976** | **661** | **988** | **1110** | **1045** |
| **Alkenes:** |  |  |  |  |  |  |  |
| 1,4,8-dodecatriene | 2343 | LRI/MS | 90 ± 1.55 | 89 ± 1.06 | 83 ± 5.74 | 72 ± 9.22 | 67 ± 0.11 |
| 2,3,4-hexatriene | 1785 | LRI/MS | 103 ± 1.16 | 87 ± 3.37 | 60 ± 3.06 | nd | 33 ± 7.31 |
| 1,3-Pentadiene | 515 | LRI/MS | nd | nd | nd | 59 ± 1.81 | 50 ± 1.33 |
| **TOTAL** |  |  | **193** | **176** | **143** | **196** | **150** |
| **Others:** |  |  |  |  |  |  |  |
| Caffeine | 1840 | LRI/MS/IS | 11 ± 0.83 | 13 ± 0.45 | 18 ± 0.66 | 21 ± 0.01 | 36 ± 0.44 |
| Limonene (Terpenes) | 1194 | LRI/MS | 56 ± 0.47 | 41 ± 1.33 | 23 ± 3.38 | nd | 31 ± 1.25 |
| Maltol | 2000 | LRI/MS | 81 ± 3.92 | 65 ± 2.25 | 72 ± 1.17 | 90 ± 2.43 | 48 ± 0.76 |
| Thiazole | 1253 | LRI/MS | 122 ± 1.51 | 125 ± 1.10 | 96 ± 3.88 | 85 ± 6.58 | 105 ± 3.17 |
| 2-Thiophene methanol (Thiophene) | 1921 | LRI/MS | nd. | 15 ± 0.03 | nd | 17 ± 0.13 | nd |
| 2-acetylpyrrole (N-heterocyclic) | 1960 | LRI/MS | 41 ± 0.21 | 39 ± 0.52 | nd | nd | nd |
| Acetoin | 1300 | LRI/MS | 37 ± 4.45 | 39 ± 0.18 | 44 ± 2.19 | 47 ± 2.81 | 18 ± 1.24 |
| Cyclopentene derivative | 1860 | LRI/MS | nd | nd | 25 ± 0.73 | 34 ± 1.52 | nd |
| **TOTAL** |  |  | **348** | **337** | **276** | **346** | **250** |

^a^, LRI (Linear Retention Index) on a DB-wax column was calculated in relation to the retention time of *n*-alkane (C_8_–C_30_) series. ^b^, Identification method, nd, not detected. Concentration of volatile compounds are presented as the mean value ± standard deviation (mean ± SD).
